# A Neuromuscular Electrical Stimulation (NMES) and robot hybrid system for multi-joint coordinated upper limb rehabilitation after stroke

**DOI:** 10.1186/s12984-017-0245-y

**Published:** 2017-04-26

**Authors:** Wei Rong, Waiming Li, Mankit Pang, Junyan Hu, Xijun Wei, Bibo Yang, Honwah Wai, Xiaoxiang Zheng, Xiaoling Hu

**Affiliations:** 1 Interdisciplinary Division of Biomedical Engineering, The Hong Kong Polytechnic University, Hung Hom, Hong Kong; 2 Industry Centre, The Hong Kong Polytechnic University, Hung Hom, Hong Kong; 3 Qiushi Academy for Advanced Studies, Zhejiang University, Hangzhou, China; 4 Institute of Textile & Clothing, The Hong Kong Polytechnic University, Hung Hom, Hong Kong; 5 The Department of Logistics and Maritime Studies, The Hong Kong Polytechnic University, Hung Hom, Hong Kong

**Keywords:** Stroke, Robot, Neuromuscular electrical stimulation

## Abstract

**Background:**

It is a challenge to reduce the muscular discoordination in the paretic upper limb after stroke in the traditional rehabilitation programs.

**Method:**

In this study, a neuromuscular electrical stimulation (NMES) and robot hybrid system was developed for multi-joint coordinated upper limb physical training. The system could assist the elbow, wrist and fingers to conduct arm reaching out, hand opening/grasping and arm withdrawing by tracking an indicative moving cursor on the screen of a computer, with the support from the joint motors and electrical stimulations on target muscles, under the voluntary intention control by electromyography (EMG). Subjects with chronic stroke (*n* = 11) were recruited for the investigation on the assistive capability of the NMES-robot and the evaluation of the rehabilitation effectiveness through a 20-session device assisted upper limb training.

**Results:**

In the evaluation, the movement accuracy measured by the root mean squared error (RMSE) during the tracking was significantly improved with the support from both the robot and NMES, in comparison with those without the assistance from the system (*P* < 0.05). The intra-joint and inter-joint muscular co-contractions measured by EMG were significantly released when the NMES was applied to the agonist muscles in the different phases of the limb motion (*P* < 0.05). After the physical training, significant improvements (*P* < 0.05) were captured by the clinical scores, i.e., Modified Ashworth Score (MAS, the elbow and the wrist), Fugl-Meyer Assessment (FMA), Action Research Arm Test (ARAT), and Wolf Motor Function Test (WMFT).

**Conclusions:**

The EMG-driven NMES-robotic system could improve the muscular coordination at the elbow, wrist and fingers.

**Trial registration:**

ClinicalTrials.gov. NCT02117089; date of registration: April 10, 2014

## Background

Stroke is a main cause of long-term disability in adults [1]. Approximately 70 to 80% stroke survivors experienced impairments in their upper extremity, which greatly affects the independency of their daily living [2, 3]. In the upper limb rehabilitation, it also has been found that the recovery of the proximal joints, e.g., the shoulder and the elbow, is much better than the distal, e.g., the wrist and fingers [4, 5]. The main possible reasons are: 1) The spontaneous motor recovery in early stage after stroke is from the proximal to the distal; and 2) the proximal joints experienced more effective physical practices than the distal joints throughout the whole rehabilitation process, since the proximal joints are easier to be handled by a human therapist and are more voluntarily controllable by most of stroke survivors [2]. However, improved proximal functions in the upper limb without the synchronized recovery at the distal makes it hard to apply the improvements into meaningful daily activities, such as reaching out and grasping objects, which requires the coordination among the joints of the upper limb, including the hand. More effective rehabilitation methods which may benefit the functional restoration at both the proximal and the distal are desired for post-stroke upper limb rehabilitation.

Besides the weakness and spasticity of muscles in the paretic upper limb, discoordination among muscles is also one of the major impairments after stroke, mainly reflected as abnormal muscular co-activating patterns and loss of independent joint control [2, 6]. Stereotyped movements of the entire limb with compensation from the proximal joints are commonly observed in most of persons with chronic stroke who have passed six months after the onset of the stroke, during which abnormal motor synergies were gradually developed. Neuromuscular electrical stimulation (NMES) is a technique that can generate limb movements by applying electrical current on the paretic muscles [7]. Post-stroke rehabilitation assisted with NMES has been found to effectively prevent muscle atrophy and improve muscle strength [7], and the stimulation also evokes sensory feedback to the brain during muscle contraction to facilitate motor relearning [8]. It has been found that NMES can improve muscular coordination in a paralysed limb by limiting ‘learned disuse’ that stroke survivors are gradually accustomed to managing their daily activities without using certain muscles, which has been considered as a significant barrier to maximizing the recovery of post-stroke motor function [9]. However, difficulties have been found in NMES alone to precisely activate groups of muscles for dynamic and coordinated limb movements with desired accuracy in kinematics, for example, speeds and trajectories. It is because most of the NMES systems adopted transcutaneous stimulation with surface electrodes only recruiting muscles located closely to the skin surface with limited stimulation channels [8]. Therefore, the muscular force evoked may not be enough to achieve the precise limb motions. However, limb motions with repeated and close-to-normal kinematic experiences are necessary to enhance the sensorimotor pathways in rehabilitation, which has been found to contribute to the motor recovery after stroke [10]. Furthermore, faster muscular fatigue would be experienced when using NMES with intensive stimuli, in comparison with the muscle contraction by biological neural stimulation [11].

The use of rehabilitation robots is one of the solutions to the shortage of affordable professional manpower in the industry of physical therapy, to cope with the long-term and labour-demanding physical practices [10]. In comparison with the NMES, robots can well control the limb movements with electrical motors. Various robots have been proposed for upper limb training after stroke [12, 13]. Among them, the robots with the involvement of voluntary efforts from persons after stroke demonstrated better rehabilitation effects than those with passive limb motions, i.e., the limb movements are totally dominated by the robots [10]. Physical training with passive motions only contributed to the temporary release of muscle spasticity; whereas, voluntary practices could improve the motor functions of the limb with longer sustainability [10, 14]. In our previous studies, we designed a series of voluntary intention-driven rehabilitation robotics for physical training at the elbow, the wrist and fingers [14–18]. Residual electromyography (EMG) from the paretic muscles was used to control the robots to provide assistive torques to the limb for desired motions. The results of applying these robots in post-stroke physical training showed that the target joint could obtain motor improvements after the training; however, more significant improvements usually appeared at its neighbouring proximal joint mainly due to the compensatory exercises from the proximal muscles [15, 17]. In order to improve the muscle coordination during robot-assisted training, we integrated NMES into the EMG-driven robot as an intact system for wrist rehabilitation [16, 19]. It has been found that the combined assistance with both robot and NMES could reduce the excessive muscular activities at the elbow and improve the muscle activation levels related to the wrist, which was absent in the pure robot assisted training [16]. More recently, combined treatment with robot and NMES for the wrist by other research group also demonstrated more promising rehabilitation effectiveness in the upper limb functions than pure robot training [20]. However, most of the proposed devices are for single joint treatment, and cannot be used for multi-joint coordinated upper limb training. Furthermore, the training tasks provided by these devices are not easy to be directly translated into daily activities. We hypothesized that multi-joint coordinated upper limb training assisted by both NMES and robot could improve the muscular coordination in the whole upper limb and promote the synchronized recovery at both the proximal and distal joints. In this work, we designed a multi-joint robot and NMES hybrid system for the coordinated upper limb physical practice at the elbow, wrist and fingers. Then, the rehabilitation effectiveness with the assistance of the device was evaluated by a pilot single-group trial. EMG signals from target muscles were used for voluntary intention control for both the robot and NMES parts.

## Methods

### The NMES-robot system

**Fig. 1:**
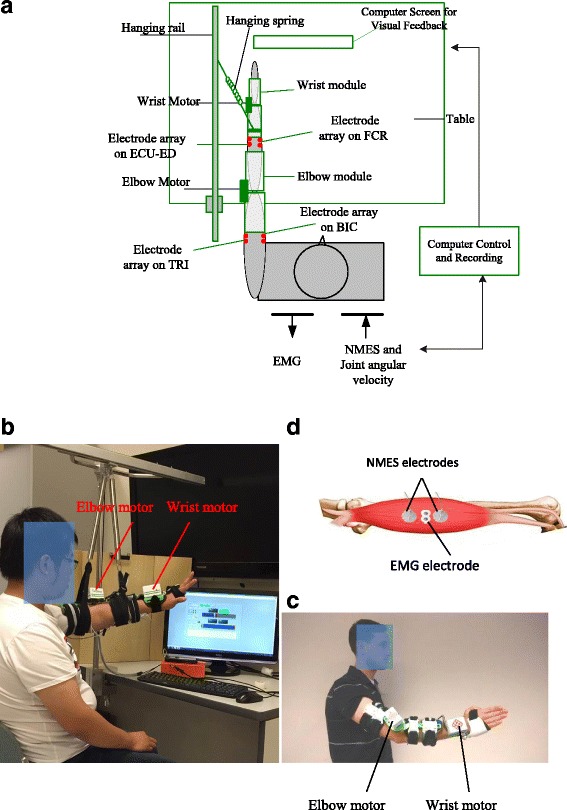
**a** The schematic diagram of the experimental setup, **b** a photo of a subject who is conducting the tracking task with the NMES-robot, **c** a photo of a subject wearing the mechanical parts of the system, **d** the configuration of the NMES electrodes and EMG electrodes on a driving muscle. The driving muscles in the study are BIC, TRI, FCR and the muscle union of ECU-ED

The system developed is a wearable device as shown in Fig. 1. It can support a stroke subject to perform sequencing limb movements, i.e., 1) elbow extension, 2) wrist extension associated with hand open, 3) wrist flexion and 4) elbow flexion, with the purpose of simulating the coordination of the joints in arm reaching out, hand open for grasping, and withdrawing in daily activities. The starting position of the motion cycle was set at the elbow joint extended at 180° and the wrist extended at 45°, which is also the end point for a motion cycle. In each phase of the motion, visual guidance on a computer screen was provided to a subject by following a moving cursor on the computer screen with a constant angular velocity at 10°/s for the movement of the wrist and the elbow. The subject was asked to minimize the target and actual joint positions during the tracking. In the limb tasks, assistances would be provided from the mechanical motors and NMES at the same time related to the wrist and elbow flexion/extension. NMES alone was applied for finger extension, and there was no assistance from the system for finger flexion (hand grasp). It is because that the main impairment in the hand for persons with chronic stroke is hand open, and the hand grasp can be achieved passively due to spasticity in finger flexors, and one channel NMES has demonstrated the capacity to achieve the gross open of the hand with finger extensions in clinical practices [2]. With the attempt to reduce the overall weight of the system, especially at the distal joints, for the coordinated multi-joint training of the whole upper limb, finger motions were only supported by the NMES in this work. The robot and NMES combined effects on individual finger motions in chronic stroke have been investigated in our previous work [21]. A hanging system was used to lift up the testing limb to a horizontal level (Fig. 1), to compensate the limb gravity and the weight of the wearable part of the system (totally 895 g).

The mechanical part of the system is an exoskeleton with two modules, i.e., the elbow module and the wrist module. Each of them has two orthotic limb extensions connected with a motor joint (MX 106, ROBOTIS, with a maximal stall torque of 8.4 Nm). The two modules are not mechanically connected, with the purpose to fit for subjects with different ergonomic parameters, such as limb length and pronation angles away from the neural position at the wrist in stroke subjects mainly due to joint stiffness and muscle spasticities after stroke [22]. The two modules can be fixed to the respective joints by a bracing system with adaptive control on the pressure applied to the skin, which can minimize the migration of the device during repeated limb movements [23]. In the wrist module, the orthosis only covers the palm at the hand side, but leaves the fingers free for flexion and extension. The maximum range of motion (ROM) for the elbow controlled by the motor was from 30° of elbow flexion to 180° of elbow extension in this study. For the wrist joint, the maximum ROM was from 45° of the extension (denoted as the negative) to 60° of the flexion (denoted as the positive). The ROMs for the elbow and wrist joints had been tested on their feasibilities when applied to stroke subjects in our previous works [15, 16]. Four-channel NMES was applied on the muscles of biceps brachii (BIC) during elbow flexion, triceps brachii (TRI) during elbow extension, flexor carpi radialis (FCR) for wrist flexion, and the last channel on both the extensor carpi ulnaris (ECU) and extensor digitorum (ED) for wrist extension and the associated hand open (i.e., finger extension). The muscles of the ECU and ED are close to each other anatomically with narrow muscle bellies on the dorsal side of the forearm, and can be recruited together by just one-channel surface NMES [24]. They were treated as a muscle union (ECU-ED) for both NMES and EMG detection in this work. The function of the motors and NMES are under the control of the EMG detected from the BIC, TRI, FCR and ECU-ED muscles. The configuration for the EMG and NMES electrodes on a target muscle (i.e., BIC, TRI and FCR in this work) is shown in Fig. 1.d, which also has been adopted in our previous NMES-robot system for wrist rehabilitation [19]. The EMG electrode pair and the NMES electrode pair were placed perpendicularly to each other on a target muscle, which was an empirical configuration to have relatively low stimulation artifact during EMG recording [19]. The common mode noise in EMG detection was minimized by the reference input to the amplifier with the electrode attached at the skin surface of the olecranon. For the ECU-ED muscle union, the EMG and NMES electrodes are located on the common area of the muscle bellies of the two.

The system can help a subject to perform the sequential and phasic motions, i.e., elbow extension, wrist extension & hand open, wrist flexion and elbow flexion in the tracking task by following the moving cursor on the screen. In each motion phase, only the EMG from the driving muscle was used for the control of the motor in the robot and the NMES applied on the same driving muscle, i.e., TRI in the elbow extension phase, ECU-ED in the wrist extension & hand open, FCR in wrist flexion and BIC in the elbow flexion; but EMG signals from the other muscles were not used in the control in the phase. This design was try to help the subject learn the association between the desired muscle contraction and the resulted limb motion assisted by both NMES (evoked the muscle contraction and indicated the location of the driving muscle) and the robot (supported the limb motion). However, the system would not give response/reward to the muscle activities of the other muscles. The outputs of a channel of the NMES are square pulses with constant amplitude of 80 V, inter-pulse interval at 25 ms (i.e., stimulation frequency of 40 Hz), and varied pulse width from 0 to 200 μs. The Assistance from the NMES is defined as

1 $$ {W}_{NMES, phase}(t)={W}_{i, max}\cdot \overline{M}{(t)}_{i,\  phase} $$

where, W_NMES, phase_(t) is the real-time stimulation pulse width in a motion phase applied to a driving muscle, i.e., the respective elbow extension, wrist extension & hand open, wrist flexion and elbow flexion in the tracking task. W_i,max_ is the maximum stimulation impulse width applied on the target driving muscle i. It is the threshold values to evoke visible elbow flexion and extension, maximal wrist extension adjunct with full finger extension, and maximal wrist flexion, when the upper limb is positioned horizontally with the hanging system. This setting of the stimulation intensity could be accepted by most of chronic stroke subjects during multiple session training in aspects of the muscle fatigue and the pain sensation experienced individually during the stimulation in our previous study; and the sensation achieved during the stimulation also could indicate the location of a target muscle to the stroke subject [16, 19, 21]. In Eq (1), M(t)_i,phase_ is defined as

2 $$ M{(t)}_{i, phase}=\frac{EM{G}_i(t)- EM{G}_{i Rest}}{EM{G}_{i Max}- EM{G}_{i Rest},} $$

where EMG_i_(t) is the real time normalized EMG level of the agonist muscle, i, in its contraction phase during the tracking; EMG_iRest_ is the averaged EMG of the muscle, i, in the resting state; and EMG_iMax_ is the maximal EMG value of the muscle, i, during its isometric maximum voluntary contractions (IMVCs). The reason for the EMG normalization with respect to the value during IMVC was to minimize the effect caused by the variation in EMG electrodes applied to a muscle in multiple training sessions in a rehabilitation program [25]. The method for the EMG measurement during IMVCs would be detailed later. $\overline{M}{(t)}_{i, phase}$ in Eq 1 is the mean value of M(t)_i,phase_ during the past 25 ms (i.e., the duration between two neighbouring stimulation pulses). All EMG signals were amplified with a gain of 1000 (amplifier: INA 333, Texas Instruments Inc.), band-pass filtered from 10 to 500 Hz, and then sampled with 1000 Hz for digitization. A self-programmed sample-and-hold software (S/H) was applied for removing the stimulation artifacts after the EMG signals were digitized. The similar methods have been adopted in our previous NMES-robot for wrist rehabilitation [19, 26]. Finally, the EMG signals were full-wave rectified and moving-averaged with 100 ms window to obtain the EMG levels (i.e., EMG_i_(t)) before input into Eq 2.

The movement speeds of the motors are also under the control of the EMG signals from the driving muscles. The angular velocity of a motor is defined as follows:

3 $$ {\theta}_{phase}(t)={\theta}_{M ax}\cdot \overline{M}{(t)}_{i,\  phase}, $$

where, θ_phase_(t) is the real-time angular velocity of the motor in either flexion or extension phase of the related joint. θ_Max_ is a preset maximal joint angular velocity, and 30°/s was selected in this study, according to our experiences on the acceptability by stroke subjects for tracking tasks in previous studies [16, 19]. $\overline{M}{(t)}_{i, phase}$ has the same meaning as in Eq 2. For example, during the elbow extension phase, the maximal angular velocity provided by the elbow motor was 30°/s when the TRI EMG level reached to its maximal level, i.e., EMG_TRI,Max_; while the minimum velocity would be zero, if there was no above resting level EMG detected from the triceps muscle. Meanwhile, NMES would be delivered to the TRI with the intensity governed by Eq 1. During the tracking task, the body trunk of a subject was not constrained in this work, although previous study by Levin et al. indicated that stroke subjects would exert excessive trunk motion during reaching movement to accomplish the task goal [27]. It was because that the compensatory motions/muscle activities from other body parts would not contribute to the control of the system, and the tracking tasks designed was to minimize the difference between the target joint position and the actual position in this study, rather than researching out for a physical object as in [27].

### Evaluation on joint movement assisted by the NMES-robotic arm

**Fig. 2:**
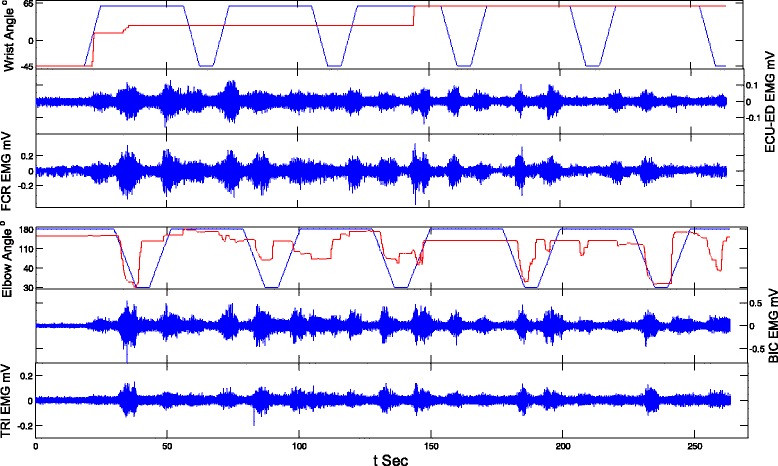
The representative EMG trials and tracking trajectories (The *blue lines* are for the target positions and the *red lines* are for the actual positions) when there was no assistance from the NMES-robotic arm (N0R0)

The assistive capacity of the designed NMES-robot arm was evaluated by four different assistive schemes as shown in Table 1, with the purpose of understanding the different contributions from the robot and NMES to the upper limb movements of stroke subjects. After obtaining the approval from the Human Subjects Ethics Sub-Committee of the Hong Kong Polytechnic University, 11 persons after stroke were recruited from different districts in Hong Kong through advertisement. Written informed consents were obtained from all recruited subjects in this study. The demographic data of the recruited stroke subjects is shown in Table 2. The inclusion criteria were 1) the subjects were at least one year after the onset of a singular and unilateral brain lesion due to stroke; 2) the passive ROM of the subjects for the wrist was from −45 to 60° and the ROM for the elbow was from 30 to 180°; 3) the spasticity at the elbow, the wrist and the fingers were below 3 as measured by the Modified Ashworth Scale (MAS, ranged from 0 (no increase in the muscle tone) to 4 (affected part rigid)) [28], 4) motor impairments in the upper limb were severe to moderate as assessed by Fugl-Meyer Assessment (15 < FMA < 45, with a maximal score of 66) [29], 5) the subjects had no vision impairment and could follow the instruction of the training protocol, and 6) there were detectable EMG signals from the target muscles in the upper limb (i.e., 3 times of the standard deviation above the baseline). For most chronic stroke survivors with moderate to severe impairments, they can exert EMG in a target muscle, but usually accompanied with co-contractions from other muscles due to compensatory motions and discoordination of the related muscle groups [2, 6]. In an evaluation session, a subject was first instructed to perform the IMVCs on each target muscle when the wrist joint was positioned at 0° and the elbow joint at 90°. Two repetitions were conducted with a sustaining muscle contraction of 6 s for each. The maximal EMG level in the repetitions was selected for the system control and later offline processing. Then, the subject was required to conduct eight tracking tasks with the NMES-robotic arm by following the moving target on the screen, giving the four different assistive schemes (Table 1) with a repetition of twice. Each tracking trial contained 5 cycles of 1) wrist flexion with hand grasping, 2) elbow flexion (arm withdrawing), 3) elbow extension (arm reaching out), and 4) wrist extension with hand open. Five-minute rest between two consecutive trials was allowed to avoid muscle fatigue. The sequence of the testing trials for each subject was randomized. Figure 2 shows a representative tracking trial from a subject when the system working with R0N0 mode.

**Table 1:** Notations for the different assistive schemes from the NMES-robot arm

Notation of assistive schemes	Description
R0N0	No assistance from either the robot or the NMES
R100N100	Assistance from both the robot and the NMES
R100N0	Assistance from the robot only
R0N100	Assistance from the NMES only

**Table 2:** Demographic data of the recruited subjects

Age (Years)	No. of Subjects	Gender (Female/Male)	Years after Stroke	Hemiplegia (Left/Right)	Stroke type (Haemorrhage/Ischemia)
				No. of subjects	No. of subjects
45.4 ± 16.2	11	2/9	6 ± 6.3	6/5	2/9

The performance was evaluated by EMG co-contraction index (CI) between a pair of muscles, root mean squared error (RMSE) between the target and the actual wrist positions during the tracking. The CI value of a pair of muscle would vary from 0 to 1. A high value of CI suggests a high overlap of the EMG signal of the two muscles during contraction (i.e., contracting together); while a low value of CI suggests the two muscle can contract more independently [14–16]. The calculation method of the EMG CI between different muscle pairs could be found in our previous studies [14–16].

### NMES-robotic arm assisted upper limb training

After the evaluation, the recruited stroke subjects were also invited to attend a pilot investigation on the rehabilitation effectiveness of the device assisted upper limb training in different days. The subjects received 20 training sessions, with a training intensity of 3–5 sessions/week, finished within 7 consecutive weeks, and each session lasted 1.5 h. In each training session, the IMVCs as in the evaluation session for each target muscle were first conducted to obtain the values of *EMG*_*iMax*_ and *EMG*_*iRest*_ in Eq 2, which could minimize the effects from the deviation of EMG electrode positions across the training sessions. There were 12 tracking trials in each session, and each trial consisted of five cycles of sequential motions: 1) wrist flexion with hand grasping, 2) elbow flexion (arm withdrawing), 3) elbow extension (arm reaching out), and 4) wrist extension with hand opening. During the training, the NMES-robot worked in the R100N100 mode from trial 2 to 11, whereas, the first and the last trials were in the mode of R0N0 which served as an evaluation on tracking capability of a subject when no assistance was provided from the device. A rest of 5 min was provided to the subjects between the two consecutive trials to avoid the muscle fatigue. The training effects were assessed with the clinical assessments of FMA [29], MAS (elbow, wrist and fingers), Action Research Arm Test (ARAT) [30] and Wolf Motor Function Test (WMFT), before and after the training [31], by a blinded assessor. The improvement of the skill in tracking of the subjects was monitored by the RMSE values by the assistive schemes of R0N0 and R100N100 session-by-session.

Normality test was performed on the clinical scores, RMSEs and EMG data (co-contraction indexes) by Lilliefors method with significant level of 0.05 [32]. It showed that the EMG and RMSE samples had normal distributions (*P* < 0.05), but the normality of the clinical scores was not significant (*P* > 0.05, for the pre- and post- evaluations). Therefore, Wilcoxon test was conducted on the clinical scores, by a paired comparison on the data before and after the training for the same subject. The analyses of variance (ANOVA) with multiple factors of interest (Bonferroni post hoc test with respect to the multiple evaluation and training sessions, *t*-test for the respective wrist and elbow tracking tasks) were used to evaluate the effects of the different assistive schemes on the parameters of RMSE and muscle co-contraction indexes. The level of statistical significance was set at 0.05 in this work.

## Results

**Fig. 3:**
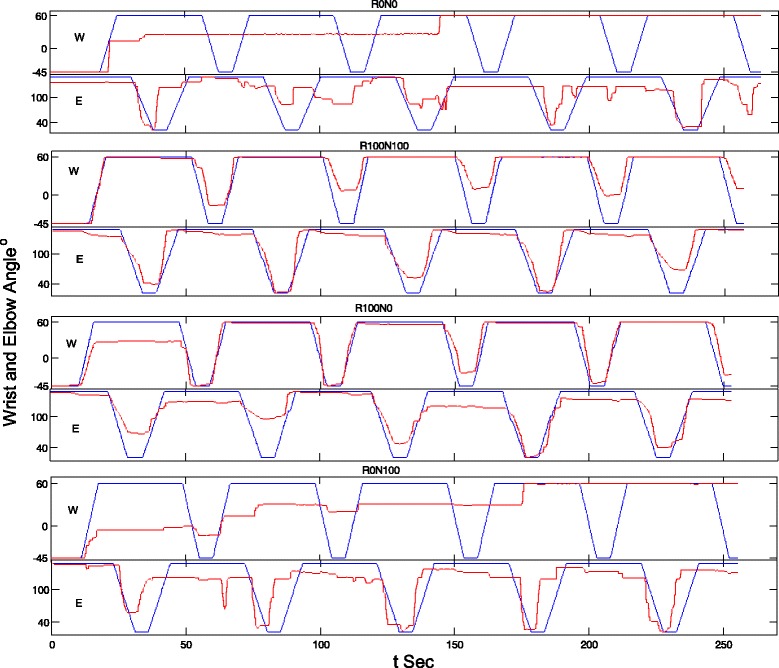
The representative tracking trajectories in the wrist (W) and the elbow (E) tracking phases, when giving different assistive schemes, i.e., 1) no assistance from either the robot or the NMES (R0N0), 2) assistance from both the robot and the NMES (R100N100), 3) assistance from the robot only (R100N0), and 4) assistance from the NMES only (R0N100). The *blue lines* are for the target positions and the *red lines* are for the actual positions

**Fig. 4:**
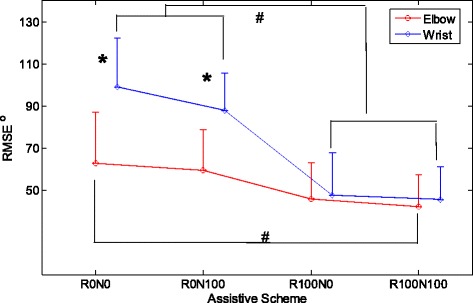
The comparison among the RMES values represented with the means (*circles and diamonds*) and standard deviations (*error bars*) across the different assistive schemes for the elbow and wrist tracking with the data from all subjects. The significant differences (*P* < 0.05) with respect to the assistive scheme are indicated by ‘#’, and the significant differences (*P* < 0.05) with respect to the joint are indicated by ‘*’

**Fig. 5:**
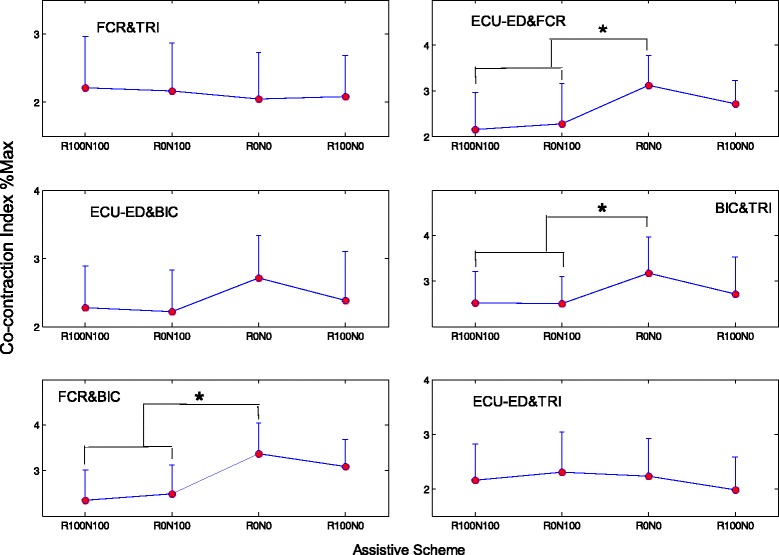
Co-contraction indexes of different muscle pairs when giving different assistive schemes from the NMES-robot arm. The CI has a unit of %Max, representing the percentage with respect to the maximum CI of 1. The significant differences (*P* < 0.05) with respect to the assistive schemes are indicated by ‘*’

Figure 3 shows the representative tracking trajectories with different assistive schemes. It could be observed that the tracking trajectories with the assistance from the robotic part (i.e., R100N100 and R100N0) were smoother than those without the robotic support (i.e., R0N100 and R0N0). Figure 4 summarized the RMSE values recorded with the different assistive schemes in the evaluation for both the wrist and the elbow joints. The RMSE varied differently with respect to the assistive schemes and to the different joints (*P* = 0.0127 and *F* = 5.83 for the factor of joint and *P* = 0.0083 and *F* = 7.13 for the assistive scheme, two-way-ANOVA). The RMSE values in wrist tracking were significant higher than the elbow when the assistive schemes were R0N100 and R0N0 (*t*-test, *P* < 0.05). However, there was no significant difference in RMSEs between the wrist and elbow tracking when the assistive schemes were R100N100 and R100N0. With the assistance from the robot part, the RMSE values (R100N100 and R100N0) were significantly lower than those without the assistance from the robot for the wrist joint (*P* < 0.001 and *F* = 21.64 One-way ANOVA, with Bonferroni post hoc test). The RMSE for R100N100 in elbow tracking was significantly lower than that with R0N0 (*P* = 0.022 and *F* = 6.11 One-way ANOVA, with Bonferroni post hoc test). Figure 5 shows the co-contraction patterns (quantified by the co-contraction index) of the muscle pairs in different assistive schemes. Significantly lowered muscle co-contractions were observed in the muscle pairs of FCR&BIC (*P* < 0.001 and *F* = 22.27, One-way ANOVA, with Bonferroni post hoc test), ECU-ED&FCR (*P* < 0.001 and *F* = 10.43, One-way ANOVA, with Bonferroni post hoc test) and BIC&TRI (*P* = 0.004 and *F* = 7.66, One-way ANOVA, with Bonferroni post hoc test) when the assistive schemes were R100N100 and R0N100.

**Fig. 6:**
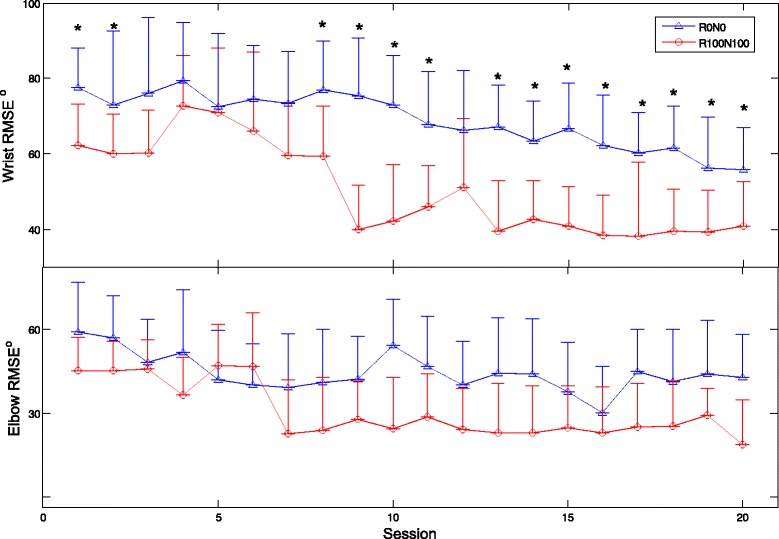
The RMSE values for the wrist and elbow tracking across the training sessions represented by the means and the standard deviations, when the assistive schemes were R0N0 and R100N100. Significant difference (*P* < 0.05) with respect to the assistive scheme are indicated by ‘*’ in the wrist tracking

Table 3 shows the clinical scores of the subjects, before and after the NMES-robot arm assisted upper limb training. Significant reduction in the MAS scores at the elbow and the wrist were observed (*P* < 0.05). However, significant MAS score reduction was absent for the fingers. Significant improvements in the FMA upper limb, ARAT and WMFT scores were also achieved after the training (*P* < 0.05). The time for conducting WMFT tasks was significantly reduced after the training (*P* < 0.05). Figure 6 shows the variations of RMSE across the training sessions. Significant decreases in the RMSE value were observed for the cases of 1) wrist R0N0 (*P* < 0.001, *F* = 8.32), 2) wrist R100N100 (*P* < 0.001, *F* = 15.6), 3) elbow R0N0 (*P* = 0.003, *F* = 5.07) and 4) elbow R100N100 (*P* < 0.001, *F* = 10.71) by One-way ANOVA with Bonferroni post hoc tests. There was no significant difference on the RMSE values with respect to the assistive schemes during the elbow tracking (*P* > 0.05, two-way ANOVA). However, the RMSE for the wrist tracking were significantly different with the assistive schemes of R0N0 and R100N100 (*P* = 0.0031, *F* = 26.7, two-way ANOVA with respect to the assistive scheme factor). The RMSE values for R100N100 were significantly lower than those of R0N0 in most of the sessions in the wrist tracking (*P* < 0.05, t-tests).

**Table 3:** Clinical scores before and after the NMES-robot arm assisted training

Score (Max value)	Pre-training	Post-training	Wilcoxon test
MAS_elbow (4)	1.51 ± 0.62	0.82 ± 0.67	*P* = 0.0045*
MAS_wrist (4)	1.65 ± 0.61	0.98 ± 0.58	*P* = 0.0012*
MAS_finger (4)	1.51 ± 0.84	0.94 ± 0.77	*P* > 0.05
FMA (66)	30.10 ± 10.19	41.0 ± 8.35	*P* = 0.0025*
ARAT (57)	19.27 ± 8.55	28.36 ± 6.46	*P* < 0.001*
WMFT (75)	36.45 ± 9.94	45.91 ± 12.42	*P* < 0.001*
WMFT_time	45.47 ± 20.44 (s)	36.19 ± 16.86 (s)	*P* = 0.037*

Significant differences (*P* < 0.05) found between the pre- and post-training assessments are indicated by ‘*’. The MAS score ‘1 + ’ was assigned a values of 1.5 as practiced in the literature [35]

## Discussion

### Evaluation on the assistive capacity of the NMES-robot arm

The NMES-robotic arm developed in this study could assist the paretic limb of stroke subjects to achieve the multi-joint and phasic movements simulating the arm reaching out with hand opening and arm withdrawing. The mobility of the wrist joint in the subjects recruited in this study was poorer than the elbow joint, which is common in persons with chronic stroke [1, 2]. For example (Fig. 3, R0N0), the subject could not perform the wrist tracking when there was no assistance from the system, and the wrist joint almost always remained in a constant flexed position for the last two tracking cycles. However, similar immobilization was absent at the elbow joint. Relatively poor performance in wrist tracking was also observed when only NMES provided the assistance (Fig. 3, R0N100), where the wrist joint was immobilized in a flexed position in the last one and half cycles. It has been found in our previous study [22] that subjects with chronic stroke usually have more difficulties in exerting voluntary EMG in the wrist extensor when the wrist joint is placed in an extremely flexed position (e.g., 60° flexed in this work and in [22]) than the other wrist joint positions, represented as a relatively low voluntary EMG level. When there was only assistance from the NMES part whose intensity was proportional to the voluntary EMG level of the wrist extensor, the voluntary effort, together with the evoked muscle force by NMES, possibly was not enough to help the wrist out of the ‘locked’ place, e.g., 60° flexed of the wrist as in Fig. 3 (R0N100). The wrist tracking performance was sensitive to the assistance from the robotic part. With the support from the robot, the tracking became smoother. As shown in Fig. 4, the wrist tracking accuracies were at the similar level as for the elbow, when robot gave the support (R100N100 and R100N0). However, the wrist tracking errors were significantly higher than the elbow, if the robotic assistance was removed (R0N100 and R0N0). It implied that more severe muscle weakness at the wrist, in particular in the extensor, contributed to the poorer tracking, in comparison to the elbow. Once the weak EMG signals related to the wrist were proportionally amplified and translated to the motor joint angular velocity, the subjects could immediately improve the tracking accuracy. It also suggested that the wrist muscles were weak, but still controllable by the subjects, and NMES alone was not enough to help the subjects to achieve the wrist tracking tasks in this study.

NMES could reduce the co-contraction between muscle pairs in the evaluation tracking tasks (Fig. 5). The reduction of the co-contraction between BIC & TRI and between ECU-ED & FCR suggested a better coordination between the flexor and extensor at the respective elbow and wrist joints, when the subjects were instructed to carry out the antigravity joint flexion and extension tasks. The reduction of the co-contraction between FCR & BIC indicated a release in the co-activating pattern of the elbow and wrist, i.e., the two joints could move more independently. The subjects did not have any learning experience on using the NMES-robot arm before the evaluation test. It also implied that NMES could immediately improve the muscle coordination at both the wrist and the elbow when conducting the multi-joint tasks. In this work, four combinations of the assistive schemes (i.e., R0N0, R100N0, R0N100 and R100N100) were evaluated with the purpose to investigate the contribution from either the NMES or the robot parts, in comparison with the joint effects when both parts provided the assistance. The optimization on the assistive scheme with fine assistive levels of each part will be investigated later to achieve better movement accuracies and muscular coordinations.

### NMES-robot assisted upper limb rehabilitation

Based on the results obtained in the evaluation session, the subjects could achieve the best tracking performance (i.e., lowest RMSE and lowest muscle co-contractions) with the assistive scheme of R100N100. Therefore, in the rehabilitation training, R100N100 was adopted for the device-assisted, intensive and repeated physical practice. After the NMES-robot assisted upper limb rehabilitation training, release of muscle spasticity was obtained at both the elbow and the wrist joints. It suggested that the combined treatment of the NMES and robot with the multi-joint practice could benefit the proximal and the distal joints at the same time. However, it was also observed that no significant reduction of muscular spasticity was achieved at the finger joints. In this work, there was only one channel of NMES applied at the ED muscle for the finger extension, and there was no additional mechanical or NMES support to the finger flexors during the limb tasks. The spasticity measured in relation to a joint mainly contributed from the flexors during passive joint motions by the assessor [6, 33]. Although all subjects recruited in this study could perform the hand grasp motion without assistance, it seems NMES on the ED only for finger practice was not enough to release the muscle tone. Boyaci et al. also reported in their randomized clinical trial that either EMG-triggered or passive NMES on finger and wrist extensors could not result in a release in finger spasticity after stroke, with a comparable training intensity at the finger joints to this work [24].

NMES-robot arm assisted multi-joint upper limb practice could improve the voluntary motor functions of the whole upper limb, as indicated by the pre- and post- FMA scores (Table 3). The common improvements in all subjects were the reduction in the tremor and dysmetria. It may imply that the tracking tasks practised in this study could improve the stability of the limb motions. Although there was no specific tracking tasks assigned to finger joints in the study, the increase in the ARAT score after the training also suggested the voluntary motor improvement at the fingers. It is because that most of the evaluation tasks in ARAT are related to voluntary finger functions, for example, grasp an object and lift it up, hold a cup of water and pull into another empty cup, pick up a rod and release, etc. [30]. The main improved items in the post-assessment were the tasks related to hand release. Before the training, most of the subjects could hold an object, but could not open the hand to release the object on a table. However, after the training, eight of the recruited subjects could successfully release an object (e.g., a rod with a diameter of 2.5 cm) on the table. It suggested that the single channel stimulation on the finger extensor improved the hand open function of the subjects. The improvement captured by WMFT score suggested that the subjects could perform better in actions close to daily tasks, such as holding a can to drink, stacking chesses (cylinder wooden blocks with a diameter of 3 cm and a height of 1.2 cm) and folding a towel, which required the muscular coordination in the whole upper limb, in particular the hand functions. The reduced time for completing the WMFT tasks indicated an increase in the speed of the motions, i.e., more efficient coordination.

The decrease in the RMSE values across the training sessions (Fig. 6) suggested that the subjects could improve the tracking accuracy at both the wrist and elbow joints through the repeated practice. To reduce the RMSE during the training, a subject needed to improve the fine control of the target driving muscles, in order to obtain necessary assistance from the device. Either over excitation or lowered EMG level of the driving muscle would increase the RMSE value (i.e., advanced or delayed). The decreased RMSEs in the wrist and elbow joints across the training sessions showed that the subjects gradually learned the skill to fine-tune the EMG levels in the target muscles by the repeated practice. The improved muscle control through the tracking tasks was also reflected as the improved stability assessed by FMA and WMFT scores and the improved timing in WMFT speed evaluation. It was also noticed that for the elbow tracking, the RMSE values were similar across the training sessions when assisted with the schemes of R0N0 and R100N100. Although significant difference in the RMSE between the two assistive schemes was obtained in the evaluation (Fig. 4), the difference was minimized in the rehabilitation training with multiple sessions for repeated practice. However, the RMSE difference in the wrist tracking related to the two assistive schemes still existed across the whole training sessions. It may imply that the muscle weakness related to the wrist movement was still severe even after the training, in comparison to the elbow joint. More training sessions could possibly improve the wrist function further. When using the system in the multi-session rehabilitation training, the subjects could gradually grasp the motor skill of exerting suitable EMG levels in a desired muscle to obtain the necessary assistance, as indicated by the significant decreases in the RMSE across the training sessions. The large RMSEs in early training sessions were mainly due to the advance of the joint position in the flexion phase (i.e., moving too fast with an excessive EMG level) and the lag behind in the extension phase (i.e., moving too slow with an inadequate EMG level), in comparison with the target indicated by the moving cursor on the screen. It was because that in most of chronic stroke survivors the spasticity is commonly observed in the flexors, while muscle weakness is usually associated with extensors [6]. It was also noticed that the mean values of RMSE with R0N0 (76.6°) and R100N100 (61.0°) at session 1 for the wrist tracking in the training (Fig. 6) varied around 30% from those detected in the evaluation session (R0N0 87.9°, R100N100 42.3°, Fig. 4). However, the variation in the RMSEs for the elbow tracking was within 10%. It suggested that the tracking performance at the wrist was not as stable as at the elbow in the early sessions. The tracking performance at the wrist was gradually stable after session 9 in the study (Fig. 6).

When using the system developed in this work, the stroke subjects needed to use a hanging system to support the upper limb, since most of the persons with chronic stroke experienced muscle atrophy at the shoulder joint [34]. They have difficulties to abduct/flex the shoulder and lift up the whole paretic upper limb to the required position in the experiment even without mounting the system. The hanging system compensated the weights of the paretic upper limb and the NMES-robot arm mounted on the limb in the training at a horizontal level. The training setup proposed could be applied to in-door clinical practice, e.g., in hospitals and clinics or even home based rehabilitation. Although there was no active actuation for the shoulder joint in the training setup, most of the subjects in this study reported a perceptible strengthening of the shoulder muscles after the training, possibly due to the lifted upper limb position when practicing the arm reaching and withdrawing motions, during which shoulder muscles were also involved. In our future works, we will investigate the activities in the shoulder muscles, together with the muscles related to the elbow, wrist and fingers. Randomized controlled trials with more subject numbers will be conducted to compare the training effects with the traditional physical/occupational training and with the rehabilitation effects achieved by single-joint systems. During the mounting of the system onto a paretic upper limb of a stroke survivor, a junior student helper with a background of rehabilitation could complete the device setup and subject preparation in 15 min in the study, provided 3 tutorial sessions with the supervision from a senior staff in the project. The training system designed in this work is a prototype for the investigation of the rehabilitation feasibility and effectiveness. Product optimization is needed to improve its outlook and user experience during the process of further commercialization, e.g., designs for convenience and comfortable wearing in long-term training and easy operation in the routine clinical practice.

## Conclusions

In this work, a new EMG-driven multi-joint NMES-robot hybrid system was developed for coordinated upper limb rehabilitation on stroke subjects. The system could improve the mobility of the wrist joint mainly by the assistance from the robotic support. The assistance from the multi-channel NMES could improve the muscle coordination in the whole upper limb, by reducing the co-contractions between the antagonist muscle pairs related to the wrist and the elbow and the co-contraction between the elbow and the wrist flexors. The 20-session device assisted upper limb rehabilitation could effectively release the muscular spasticity at the wrist and the elbow. The rehabilitation training also improved the limb stability and voluntary motor functions at the elbow, the wrist and the fingers. Large scale randomized controlled trials will be conducted in our future work to further quantified the rehabilitation effectiveness of the NMES-robot arm, in comparison with those devices for single joint training and with the effects achieved by the traditional rehabilitation training.

## Abbreviations

ARAT: Action Research Arm Test
BIC: Biceps brachii
CI: Co-contraction index
ECU: Extensor carpi ulnaris
ED: Extensor digitorum
EMG: Electromyography
FCR: Flexor carpi radialis
FMA: Fugl-Meyer Assessment
IMVC: Isometric maximum voluntary contraction
MAS: Modified Ashworth score
NMES: Neuromuscular electrical stimulation
RMSE: Root mean squared error
ROM: Range of motion
TRI: Triceps brachii
WMFT: Wolf Motor Function Test

## References

[1] Saunders DH, Sanderson M, Hayes S, Kilrane M, Greig CA, Brazzelli M, Mead GE. Physical fitness traning for stroke patients. Cochrane Database Syst Rev. 2016:doi: 10.1002/14651858.CD14003316.pub14651856. 10.1002/14651858.CD003316.pub6 PMC6464717 27010219

[2] Raghavan P (2015). Upper limb motor impairment after stroke. Phys Med Rehabil Clin N Am.

[3] Nakayama H, Jorgensen HS, Raaschou HO, Olsen TS (1994). Recovery of upper extremity function in stroke patients: the Copenhagen stroke study. Arch Phys Med Rehabil.

[4] Chae J, Yang G, Park BK, Labatia I (2002). Delay in initiation and termination of muscle contraction, motor impairment, and physical disability in upper limb hemiparesis. Muscle Nerve.

[5] Dobkin BH (2004). Strategies for stroke rehabilitation. Lancet Neural.

[6] Dewald JPA, Sheshadri V, Dawson ML, Beer RF (2001). Upper-limb discoordination in hemiparetic stroke: implications for neurorehabilitation. Top Stroke Rehabil.

[7] Knutson JS, Fu MJ, Sheffler LR, Chae J (2015). Neuromuscular electrical stimulation for motor restoration in hemiplegia. Phys Med Rehabil Clin N Am.

[8] Sujith OK (2008). Functional electrical stimulation in neurological disorders. Eur J Neurol.

[9] Chae J, Yu D (1999). Neuromuscular stimulation for motor relearning in hemiplegia. Crit Revs Phy Rehab Med.

[10] Volpe BT, Ferraro M, Lynch D, Christos P, Krol J, Trudell C, Krebs HI, Hogan N (2004). Robotics and other devices in the treatment of patients recovering from stroke. Curr Atheroscler Rep.

[11] Ibitoye MO, Estigoni EH, Hamzaid NA, Wahab AK, Davis GM (2014). The effectiveness of FES-evoked EMG potentials to assess muscle force and fatigue in individuals with spinal cord injury. Sensors.

[12] Mehrholz J, Pohl M, Platz T, Kugler J, Elsner B. Electromechanical and robot-assisted arm training for improving activities of daily living, arm function, and arm muscle strength after stroke. Cochrane Database Syst Rev. 2015:doi: 10.1002/14651858.CD14006876.pub14651854. 10.1002/14651858.CD006876.pub4 PMC6465047 26559225

[13] Chang WH, Kim YH (2013). Robot-assisted therapy in stroke rehabilitation. J Stroke.

[14] Hu XL, Tong KY, Song R, Zheng XJ, Leung WFW (2009). A comparison between electromyography-driven robot and passive motion device on wrist rehabilitation for chronic stroke. Neurorehabil Neural Repair.

[15] Hu XL, Song R, Tong KY, Tsang SF, Leung PO, Li L (2007). Variation of muscle coactivation patterns in chronic stroke during robot-assisted elbow training. Arch Phys Med Rehabil.

[16] Hu XL, Tong KY, Ho SK, Xue JJ, Rong W, Li LSW. Wrist rehabilitation assisted by an electromyography-driven neuromuscular electrical stimulation robot after stroke. Neurorehabil Neural Repair. 2015;29(8):doi: 10.1177/1545968314565510. 10.1177/1545968314565510 25549656

[17] Hu XL, Tong KY, Wei XJ, Rong W, Susanto EA, Ho SK (2013). The effects of post-stroke upper-limb training with and electromyography (EMG)-driven hand robot. J Electromyogr Kinesiol.

[18] Song R, Tong KY, Hu XL, Li L (2008). Assistive control system using continuous myoelectric signal in robot-aided arm training for patients after stroke. IEEE Trans Neural Syst Rehabil Eng.

[19] Hu XL, Tong KY, Li R, Xue JJ, Ho NS, Chen P (2012). The effects of electromechanical wrist robot assistive system with neuromuscular electrical stimulation for stroke rehabilitation. J Electromyogr Kinesiol.

[20] Lee YY, Lin KC, Cheng HJ, Wu CY, Hsieh YW, Chen CK. Effects of combining robot-assisted therapy with neuromuscular electrical stimulation on motor impairment, motor and daily function, and quality of life in patients with chronic stroke: a double-blinded randomized controlled trial. J Neuroeng Rehabil. 2015:doi: 10.1186/s12984-12015-10088-12983. 10.1186/s12984-015-0088-3 PMC4628254 26520398

[21] Rong W, Tong KY, Hu XL, Ho SK (2013). Effects of electromyography-driven robot-aided hand training with neuromuscular electrical stimulation on hand control performance after chronic stroke. Disabil Rehabil Assist Technol.

[22] Hu XL, Tong KY, Tsang SF, Song R (2006). Joint-angle-dependent neuromuscular dysfunctions at the wrist in persons after stroke. Arch Phys Med Rehabil.

[23] Hu XL, Tong KY, Hu JY, Pang MKP, Qing Y, Ng ST, Tu JB, Zhang SJ, Li WM, Rong W (2013). Wearable robotic device with bracing system with moisture and pressure management for comfortable rehabilitation, US patent application : 14093066.

[24] Boyaci A, Topuz O, Alkan H, Ozgen M, Sarsan A, Yildiz N, Ardic F (2013). Comparison of the effectiveness of active and passive neuromuscular electrical stimulation of hemiplegic upper extremities: a randomized, controlled trial. Int J Rehabil Res.

[25] Halaki M, Ginn K, Naik GR (2012). Normalization of EMG Signals: To Normalize or Not to Normalize and What to Normalize to?. Computational intelligence in electromyography analysis - a perspective on current applications and future challenges.

[26] Hu XL, Tong KY, Li R, Chen M, Xue JJ, Ho SK, Chen PN (2011). Combined Functional Electrical Stimulation (FES) and robotic system driven by user intention for post-stroke wrist rehabilitation. Biomechatronics in Medicine and Health Care.

[27] Levin MF, Michaelsen SM, Cirstea CM, Roby-Brami A (2002). Use of the trunk for reaching targets placed within and beyond the reach in adult hemiparesis. Exp Brain Res.

[28] Ashworth B (1964). Preliminary trials of carisoprodol in multiple sclerosis. Practitioner.

[29] Fugl-Meyer AR, Jaasko L, Leyman I, Olsson S, Steglind S (1975). The post-stroke hemiplegic patient. I: a method for evaluation of physical performance. Scand J Rehabil Med.

[30] Carroll D (1965). A quantitative test of upper extremity function. J Chronic Dis.

[31] Wolf SL, Catlin PA, Ellis M, Archer AL, Morgan B, Piacentino A (2001). Assessing Wolf motor function test as outcome measure for research in patients after stroke. Stroke.

[32] Mohd Razali N, Wah YB (2011). Power comparisons of Shapiro-Wilk, Kolmogorov-Smirnov, Lilliefors and Anderson-Darling tests. J Stat Model Anal.

[33] Daliri SS, Forogh B, Emami Razavi SZ, Ahadi T, Madjlesi F, Ansari NN (2015). A single blind, clinical trial to investigate the effects of a single session extracorporeal shock wave therapy on wrist flexor spasticity after stroke. Neurorehabil Neural Repair.

[34] Seneviratne C, Then KL, Reimer M (2005). Post-stroke shoulder subluxation: a concern for neuroscience nurses. Axone.

[35] Pandyan AD, Johnson GR, Price CI, Curless RH, Barnes MP, Rodgers H. A review of the properties and limitations of the Ashworth and modified Ashworth Scales as measures of spasticity. Clin Rehabil. 1999;13(373–383):doi: 10.1191/026921599677595404. 10.1191/026921599677595404 10498344

